# Bilateral External Oblique Intercostal Catheter for Post-operative Analgesia After Open Pancreaticoduodenectomy: A Case Report

**DOI:** 10.7759/cureus.47189

**Published:** 2023-10-17

**Authors:** Weng Ken Chan, Kok Wang Tan, Kok Peng Chong, Angelina Chia Chia Lim, Iskandar Khalid

**Affiliations:** 1 Anaesthesiology and Intensive Care, Hospital Canselor Tuanku Muhriz, Universiti Kebangsaan Malaysia, Kuala Lumpur, MYS; 2 Anaesthesiology and Intensive Care, Universiti Kebangsaan Malaysia Medical Centre, Kuala Lumpur, MYS

**Keywords:** enhanced recovery after surgery (eras), regional anesthesia, external oblique nerve catheter, external oblique intercostal block, whipple surgery, regional anaesthesia/analgesia, ultrasound guided regional anaesthesia

## Abstract

Open pancreaticoduodenectomy, also known as Whipple surgery, is a complex and painful procedure that requires a multi-modal analgesic approach for successful post-operative rehabilitation and recovery. While thoracic epidural analgesia (TEA) remains the gold standard for pain relief after open upper abdominal surgery, it carries many risks that may outweigh the potential benefits of the technique. Furthermore, in laparoscopic converted to open pancreaticoduodenectomy cases, post-operative placement of a thoracic epidural catheter is inconvenient to the patient due to pain and positioning. The external oblique intercostal (EOI) block is a novel method that provides somatic analgesia to the upper abdomen. We present a case of bilateral EOI block with catheter insertion for post-operative analgesia in a patient who underwent laparoscopic converted to open Whipple surgery.

## Introduction

Open pancreaticoduodenectomy or Whipple surgery is often done to remove tumours and treat conditions in the pancreas and biliary system. A large incision is made in the upper abdomen to gain access to the pancreas. This complex and painful surgery requires a multi-modal approach for successful post-operative rehabilitation and recovery. While thoracic epidural analgesia (TEA) is the standard for pain relief after open upper abdominal surgery, it carries risks of catastrophic complications such as spinal cord injury, and it is contraindicated in conditions such as sepsis and coagulopathy, which are not uncommon in this patient population [1-4]. Additionally, in cases where laparoscopic surgery is converted to open pancreaticoduodenectomy, placing a thoracic epidural catheter after surgery can be inconvenient for the patient due to pain and positioning.

The pain after Whipple surgery can be due to the affected region's parietal somatic and visceral pain. Its intensity is proportional to the extent of the incision and the number of abdominal drains placed after surgery. This is mainly due to the innervation from the anterior and lateral cutaneous nerve at the affected dermatomes. The external oblique intercostal (EOI) block is a novel method described by Elsharkawy et al. providing somatic analgesia to the upper abdomen [5]. We present a case of bilateral EOI block with catheter insertion for post-operative analgesia in a patient who underwent laparoscopic converted to open Whipple surgery. 

## Case presentation

A 70-year-old gentleman, American Society of Anesthesiologists physical status (ASA) class III, was planned for laparoscopic Whipple surgery for periampullary carcinoma. He has underlying diabetes, hypertension, ischemic heart disease, and a previous history of prostate carcinoma, to which he underwent prostatectomy seven years ago. After presenting with fever and symptoms of obstructive jaundice, he was diagnosed with acute ascending cholangitis, admitted, and started on broad-spectrum antibiotics. Endoscopic retrograde cholangiopancreatography (ERCP) revealed an oedematous ampulla of Vater, which was not amenable to cannulation. Subsequent radiological imaging revealed a periampullary lesion causing biliary obstruction. 

The patient consented to laparoscopic Whipple surgery with the possibility of intraoperative conversion to an open surgery. His pre-operative haemoglobin concentration was 11.8 g/dL, platelet count was 223 x 10^9^/L, prothrombin time (PT) was 13.2 seconds, international normalised ratio (INR) was 0.96 seconds, and activated partial thromboplastin time (aPTT) 38.3 seconds. Serum creatinine was 64.1 µmol/L, and electrolytes were within normal laboratory limits. On the day of the operation, the patient was put on standard monitoring consisting of non-invasive blood pressure, electrocardiogram, and pulse oximetry. As part of our institution's hepatobiliary enhanced recovery after surgery (HPB ERAS) protocol, advanced haemodynamic monitoring consisting of arterial blood pressure waveform analysis using a FloTrac sensor and central venous pressure were monitored using EV 1000 (Edwards Lifesciences, Irvine, United States) to guide the fluid therapy. Intravenous fentanyl, propofol, and rocuronium were given as part of anaesthetic induction, and anaesthesia was maintained with desflurane intraoperatively. Throughout the operation, the patient was haemodynamically stable, and a multi-modal analgesic regimen consisting of intravenous morphine, acetaminophen (paracetamol), and parecoxib was administered. 

However, surgical difficulty and aberrant anatomy led to the conversion to an open Whipple procedure via an upper midline incision from the xiphisternum to the umbilicus. Post-operatively, the patient was extubated and sent to the post-anaesthesia care unit (PACU) for close monitoring. In the PACU, despite being on intravenous fentanyl via a patient-controlled analgesia (PCA) pump, he still complained of severe pain, with a numerical rating scale (NRS) pain score of seven out of ten at rest and eight out of ten during movement. The decision was made for a bilateral EOI block as a rescue analgesic technique, to which the patient agreed. The block was performed bilaterally using a SonoPlex® STIM 22G 80 mm needle (Pajunk, Geisingen, Germany) and a high-frequency linear ultrasound probe under a strict aseptic technique at the anterior axillary line between the external oblique muscle and the seventh rib, with twenty ml of ropivacaine 0.2%. This led to a rapid analgesic onset, which peaked subsequently till the patient felt a pain score of four out of ten. As the pain reduced further, the patient could perform chest and limb physiotherapy as guided by a physiotherapist in the PACU. The PCA fentanyl was left on standby with the patient. 

**Figure 1:**
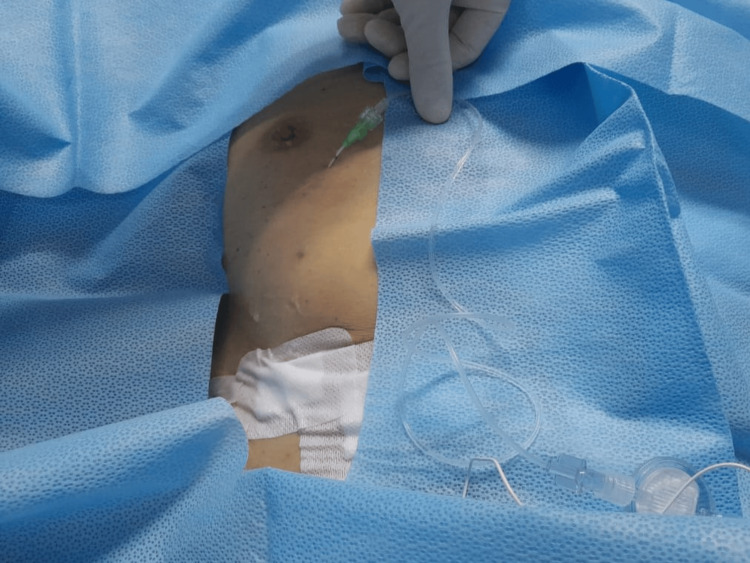
Catheter placement not interfering with the dressing site

**Figure 2:**
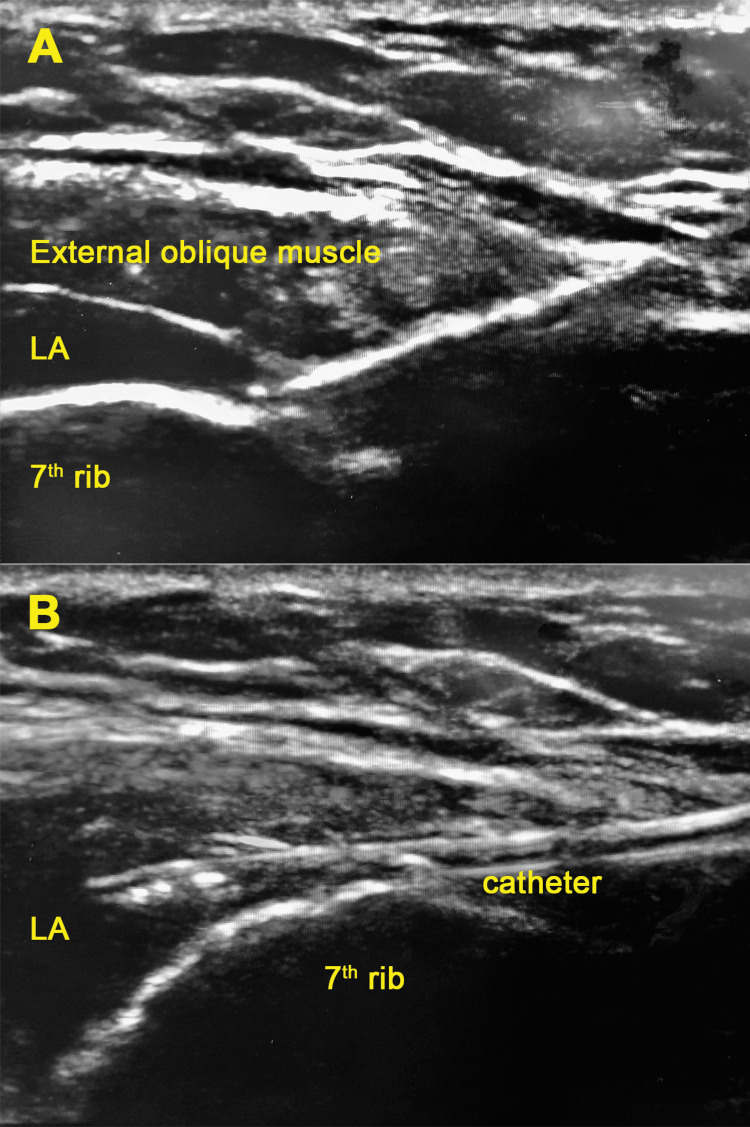
(A) Deposition of LA between the external oblique muscle and the 7th rib; (B) Placement of the catheter at the space between the external oblique muscle and the 7th rib LA: Local anaesthetic

Six hours later, the patient complained of increasing pain at the surgical site as the block effect wore off, with NRS pain score up to eight during coughing. Cumulative PCA fentanyl used up to this point was 430 mcg (delivered 43 total boluses) since it was started prior to the bilateral EOI block. After meticulous counselling, he agreed to a bilateral EOI block with catheter insertion to facilitate a prolonged analgesic effect (Figure 1). We performed the block at similar locations to the previous bilateral EOI block and with the placement of catheters bilaterally (Figure 2).

The left EOI catheter was connected to a patient-controlled pump, filled with a syringe of ropivacaine 0.1% and with a conservative setting of background infusion rate of 5 ml/hour and a patient-controlled bolus of 5 ml with a lockout time of 30 minutes. However, due to resource limitations, the right EOI catheter was connected to an infusion pump, running ropivacaine 0.1% at 5 ml/hour. His pain score reduced significantly to zero at rest and two on movement. His T6 to T12 dermatomes exhibited reduced sensation to cold. Subsequently, he was given intravenous acetaminophen and tramadol as part of a multi-modal analgesic regime. In the ward, the infusion rate of the bilateral EOI catheters was increased up to 7 ml/hour, as titrated by his pain score, with a total of 170 mcg of PCA fentanyl used since the catheter insertion. We also noted that at 5 ml/hour, the patient required more frequent additional boluses, about 70 ml over 12 hours at the left EOI catheter. This additional bolus dose was reduced to about 10 ml over 12 hours after we increased the baseline infusion to 7 ml/hour. An improvement in pain control with the bilateral EOI catheters had led to a marked reduction in PCA fentanyl usage to only 40 mcg over 24 hours and, hence, was taken off after a total of two days of usage. 

Both catheters were removed on post-operative day (POD) four, and in the following days, the patient had zero pain score at rest and only mild pain at the incision site during movement. His hospital stay was slightly prolonged due to other non-surgical complications. He had supraventricular tachycardiac (SVT) on POD five, which resolved after the cardiology team started him on amiodarone. Coincidentally, this patient also had post-operative SVT after prostatectomy previously. The patient also developed gastrointestinal bleed on POD nine, which was managed conservatively. The patient was discharged back home well subsequently on POD 17 and consented to the case report.

## Discussion

Open Whipple surgery is a complex procedure commonly performed for patients with underlying periampullary carcinoma, which carries a significant risk of morbidity and mortality due to the patient's underlying condition, surgical complexity and post-operative pulmonary complications. Hence, multidisciplinary team management is imperative to facilitate post-operative recovery for the patient [1,2].

While TEA remains the gold standard for post-operative pain control, particularly during coughing, deep breathing and chest physiotherapy, thoracic epidural insertion poses non-negligible risks, including hypotension, bradycardia, spinal cord injury, epidural hematoma, epidural abscess, inadvertent dural puncture and headache, especially in inexperienced hands [1-4]. Hence, an alternative technique with equal efficacy but lower risks would be ideal, especially in training centres that regularly perform open hepatobiliary surgery. Other conventional approaches have been described, such as the subcostal transversus abdominis plane (TAP) block and rectus sheath block, but these are not without limitations. The subcostal TAP and the rectus sheath block provide analgesia within the innervation of the anterior cutaneous nerve of the abdomen, hence missing the lateral abdomen innervated by the lateral cutaneous nerves [6]. To effectively ameliorate pain from the upper abdominal incision and drain placement after Whipple surgery, blockade of both the anterior and lateral cutaneous nerves of the upper abdomen is paramount.

The external oblique intercostal block is a novel technique described by Elsharkawy et al. to provide somatic analgesia to the upper abdomen [5]. Their case series describes sensory blockade of the anterior and lateral cutaneous nerves innervating the upper abdomen from the T6 to T10 dermatomes [5]. This technique, combined with a multi-modal analgesic regimen, can provide adequate post-operative pain relief, particularly during coughing, deep breathing and chest physiotherapy, hence minimising the risk of post-operative pulmonary complications. This block is also relatively easy to perform in the supine position and does not interfere with the surgical incision, dressing or drain site. Therefore, in cases of laparoscopic Whipple surgery converted to an open technique, bilateral EOI block with or without catheter insertion can be performed post-operatively without requiring the patient to sit up or lie laterally, unlike the thoracic epidural insertion. 

Single-shot EOI block and intermittent EOI local anaesthetic (LA) injection via catheter have been described in some articles, but literature on continuous EOI catheter technique, especially in regards to Whipple surgery, still remains limited [5, 7-11]. The optimal dosing and infusion regime has yet to be determined. In this patient, we noted that the EOI block had a rapid analgesic onset after the block was performed. The offset of the block effect was seen after six hours, which may be explained by its proximity to the intercostal vessels, which may hasten the systemic absorption of the LA. However, more research is needed to explain this finding, and the plasma level of LA should be measured to ascertain this observation. Our initial infusion regime of 5 ml/hour was increased to 7 ml/hour based on his pain score. This subsequently led to adequate analgesia and facilitated the post-operative rehabilitation program as part of our HPB ERAS protocol. With this infusion rate, we also observed a remarkable reduction in the patient-controlled bolus LA dose requirement, and this infusion of 14 ml per hour of 0.1% ropivacaine is below the maximum recommended weight-based dosing range for this local anaesthetic. 

The EOI block with continuous catheter infusion has the potential to offer effective pain relief with minimal risk to the patient. However, randomised controlled trials with larger sample sizes are needed to ascertain its non-inferiority to TEA, which remains the gold standard for post-operative analgesia in open Whipple surgery.

## Conclusions

The bilateral EOI block with continuous catheter infusion provided effective post-operative analgesia to our patient with minimal side effects. This technique was instrumental in helping our patient achieve enhanced recovery after open Whipple surgery and minimising the risk of post-operative pulmonary complications. It is a viable option for rescue analgesia and may be a suitable alternative to thoracic epidural anaesthesia, especially in patients with contraindications to inserting an epidural.
